# Coping with strong translational noncrystallographic symmetry and extreme anisotropy in molecular replacement with *Phaser*: human Rab27a

**DOI:** 10.1107/S2059798318017825

**Published:** 2019-02-28

**Authors:** Mostafa Jamshidiha, Inmaculada Pérez-Dorado, James W. Murray, Edward W. Tate, Ernesto Cota, Randy J. Read

**Affiliations:** a Faculty of Natural Sciences, Department of Life Sciences, Imperial College London, Exhibition Road, South Kensington, London SW7 2AZ, England; b Department of Chemistry, Molecular Sciences Research Hub, Imperial College London, Wood Lane, London W12 0BZ, England; c Cambridge Institute for Medical Research and Department of Haematology, University of Cambridge, Wellcome Trust/MRC Building, Hills Road, Cambridge CB2 0XY, England

**Keywords:** molecular replacement, *Phaser*, anisotropy, translational noncrystallography symmetry, Rab27a, information content

## Abstract

Data pathologies caused by effects such as diffraction anisotropy and translational noncrystallographic symmetry (tNCS) can dramatically complicate the solution of the crystal structures of macromolecules. Such problems were encountered in determining the structure of a mutant form of Rab27a, a member of the Rab GTPases. Mutant Rab27a constructs that crystallize in the free form were designed for use in the discovery of drugs to reduce primary tumour invasiveness and metastasis. One construct, hRab27a^Mut^, crystallized within 24 h and diffracted to 2.82 Å resolution, with a unit cell possessing room for a large number of protein copies. Initial efforts to solve the structure using molecular replacement by *Phaser* were not successful. Analysis of the data set revealed that the crystals suffered from both extreme anisotropy and strong tNCS. As a result, large numbers of reflections had estimated standard deviations that were much larger than their measured intensities and their expected intensities, revealing problems with the use of such data at the time in *Phaser*. By eliminating extremely weak reflections with the largest combined effects of anisotropy and tNCS, these problems could be avoided, allowing a molecular-replacement solution to be found. The lessons that were learned in solving this structure have guided improvements in the numerical analysis used in *Phaser*, particularly in identifying diffraction measurements that convey very little information content. The calculation of information content could also be applied as an alternative to ellipsoidal truncation. The post-mortem analysis also revealed an oversight in accounting for measurement errors in the fast rotation function. While the crystal of mutant Rab27a is not amenable to drug screening, the structure can guide new modifications to obtain more suitable crystal forms.

## Abstract 2

The solution of a structure of human Rab27a suffering from severe anisotropy and translational noncrystallographic symmetry was aided by identifying diffraction measurements with low information content.

## 1. Introduction   

Accounting rigorously for the effects of errors in a statistical model can dramatically enhance the sensitivity of likelihood-based methods. For instance, in molecular-replacement (MR) calculations, *Phaser* (McCoy *et al.*, 2007 ▸) is able to account for the effects of errors in both the search model and in the measured diffraction data; this is difficult to achieve with methods based on the properties of the Patterson function or on the computation of correlation coefficients. In addition, information obtained from already placed search components significantly improves the signal in rotation and translation searches for subsequent components, as measured by the log-likelihood gain (LLG) and *Z*-scores (McCoy, 2007 ▸; Storoni *et al.*, 2004 ▸; McCoy *et al.*, 2005 ▸).

This sensitivity is a double-edged sword, as likelihood-based methods are also highly sensitive to defects in their statistical models. For this reason, in crystallographic applications it is essential to account for the statistical effects of anisotropy (McCoy *et al.*, 2007 ▸) and translational noncrystallographic symmetry (tNCS; Sliwiak *et al.*, 2014 ▸). The likelihood targets in versions of *Phaser* since v.2.5.4 account for the statistical effects of tNCS arising from translations combined with small changes in conformation and orientation differences up to 10°. These yield tNCS correction parameters describing changes in the expected intensities (and their probability distribution). Automated algorithms in *Phaser* can deal with simple cases of tNCS, for instance a single tNCS vector between two groups of molecules, but manual intervention by the user can be required for more complex situations, which includes a complete understanding of the cell content and identifying the tNCS vectors between the molecules (Sliwiak *et al.*, 2014 ▸).

One consequence of the intensity modulations introduced by significant anisotropy and/or tNCS is that there are bound to be systematically weak intensities with relatively large measurement errors, regardless of any overall resolution limit applied to the data. In these circumstances, it is particularly important to account rigorously for the effects of intensity-measurement error, for instance with the log-likelihood gain on intensities (LLGI) target (Read & McCoy, 2016 ▸). Problems encountered in solving the structure of Rab27a have highlighted the importance of these issues.

Rab27a is a small GTPase belonging to the large family of Ras-related in brain (Rab) proteins. Rab27a is part of the secretory pathway involved in the transport of melanosomes (Strom *et al.*, 2002 ▸) and the secretion of vesicles containing insulin (Yamaoka *et al.*, 2015 ▸), histamine (Goishi *et al.*, 2004 ▸), chemokines, matrix metalloproteases (MMPs) and exosomes (Fukuda, 2013 ▸; Brozzi *et al.*, 2012 ▸; Ostrowski *et al.*, 2010 ▸). In humans, Rab27a is overexpressed in multiple types of cancer, including breast (Wang *et al.*, 2008 ▸), lung (Li *et al.*, 2014 ▸), pancreatic (Wang *et al.*, 2015 ▸) and liver (Dong *et al.*, 2012 ▸) cancers.

Evidence supporting the role of human Rab27a (hRab27a) in multiple cancer types suggests that the inhibition of this GTPase could be a target for cancer therapy. Therefore, structural characterization of Rab27a is required for the development of specific inhibitors. Crystallographic structures of mouse Rab27a and Rab27b (mRab27a and mRab27b) in complex with the human Slp2a and Slac2a (hSlp2a and hSlac2a) effectors have been reported (Kukimoto-Niino *et al.*, 2008 ▸; Chavas *et al.*, 2008 ▸). Potential ligandable sites are located at or near the mRab27–hSlp2a and mRab27–hSlac2a interfaces, and therefore these complexes cannot be used for the characterization of Rab27a–ligand complexes. While the crystallization of Rab27a on its own would be the ideal solution to this problem, this has been unsuccessful for the human and mouse homologues (Chavas *et al.*, 2010 ▸). We therefore generated hRab27a mutants that were capable of crystallizing in the absence of effectors and were suitable for ligand-binding studies. Point mutations in hRab27a were made based on the crystal packing of mouse Rab3, the highest identity hRab27a homologue with known structure (Dumas *et al.*, 1999 ▸). This led to a construct, referred to as hRab27a^Mut^, that is able to form crystals that diffract to a maximum resolution of 2.82 Å and with the potential ligand-binding sites exposed. A complete description of the design of these mutants will be reported elsewhere.

Initial attempts to solve the structure by MR using *Phaser* (McCoy *et al.*, 2007 ▸) were unsuccessful. Inspection of the X-ray data showed that these crystals were highly anisotropic, the native Patterson function indicated strong translational non­crystallographic symmetry (tNCS) and a high copy number was predicted per asymmetric unit.

Here, we describe the solution of this difficult MR problem, as well as the improvements that the experience has inspired in *Phaser*. Moreover, the crystal structure has given us directions for further improvements in the design of Rab27a constructs that crystallize in the free form suitable for ligand discovery, which will be reported in detail elsewhere.

## 2. Materials and methods   

### 2.1. Protein production   

The cDNA template for hRab27a (UniProt code P51159) was kindly provided by Dr Miguel Seabra (Imperial College London). A gene corresponding to residues 1–192 was amplified from this cDNA and cloned into the pET-15b plasmid, generating the pET-15b-*rab27a* construct. The construct contains an N-terminal His tag followed by a Tobacco etch virus (TEV) protease cleavage site. PCR amplification was performed using Q5 High-Fidelity DNA Polymerase (New England Biolabs; NEB); the oligonucleotides 5′-CGGCTCATATGTCTGATGGAGATTATGATTAC-3′ and 5′-CGGCTGGATCCTCAGGACTTGTCCACACTCC-3′ were used as the forward and reverse primers, respectively. A Q5 Site-Directed Mutagenesis Kit (NEB) was used to introduce several mutations (Q105E, Q118K, M119T, Q140E, K144A, E145A, E146A, I149R, A150Q and K154H; the Arg50–His69 loop was replaced with the sequence TIYRN­DKRIK) in the pET-15b-*rab27a* construct to generate the pET-15b-*hrab27amut* construct. A Q78L mutation was introduced to decrease the GTPase activity of the protein, and C123S and C188S mutations were used to avoid aggregation during protein preparation. A glycine would remain as the initial residue after tag removal using TEV protease.

For the production of hRab27a^Mut^, the pET-15b-*hrab27amut* construct was transformed into *Escherichia coli* BL21 (DE3) cells (NEB). The bacteria were grown in lysogenic broth (LB) at 37°C to an OD at 600 nm of 0.6–0.8, and protein expression was then induced with 0.5 m*M* isopropyl β-d-1-thiogalactopyranoside (IPTG) at 37°C for 3 h. The cells were harvested by centrifugation at 4000 rev min^−1^ for 10 min at room temperature. The cell pellets were resuspended in 50 m*M* Tris–HCl pH 8.0, 500 m*M* NaCl, 5 m*M* MgCl_2_ (buffer *A*) supplemented with 10 m*M* imidazole. The cells were lysed with a cell disruptor (Constant Systems) at 172 MPa and centrifuged at 15 000 rev min^−1^ for 45 min at 4°C. The supernatant was loaded onto an Ni–NTA affinity column (Qiagen) equilibrated in buffer *A* supplemented with 10 m*M* imidazole. The resin was washed with 20 volumes of buffer *A* with 10 m*M* imidazole, and the protein was then eluted in buffer *A* with 300 m*M* imidazole. The protein was dialyzed against buffer *B* (50 m*M* Tris–HCl pH 8.0, 100 m*M* NaCl, 5 m*M* MgCl_2_) and the His tag was removed by overnight incubation with TEV protease (His-tagged) at a molar ratio of 1:20 in buffer *B* supplemented with 1 m*M* DTT at 4°C. DTT was removed by dialysis against buffer *B* and the protein was reloaded onto an Ni–NTA column to remove TEV protease and uncleaved protein. The purity was assessed by SDS–PAGE. The protein concentration was determined by UV–Vis absorption at 280 nm using a Nanodrop spectrophotometer (ThermoFisher).

The locked-active (GTP-bound) form of hRab27a^Mut^ was obtained by loading the protein with the nonhydrolysable GTP analogue GppNHp (Jena Bioscience). GppNHp was loaded by overnight incubation of 10 mg hRab27a^Mut^ with 25 units of Antarctic Phosphatase (NEB) in buffer *B* with 1 m*M* zinc chloride, 0.2 *M* ammonium sulfate and a fourfold molar excess of GppNHp in a final reaction volume of 2 ml at 4°C. The GTPase was further purified by size-exclusion chromatography with a Superdex 75 HiLoad (10/30) column (GE Healthcare) equilibrated in 20 m*M* Tris–HCl pH 8.0, 150 m*M* NaCl, 5 m*M* MgCl_2_. The eluted protein was concentrated to 25 mg ml^−1^ and flash-frozen in liquid nitrogen for storage.

### 2.2. Crystallization and X-ray data collection   

Sitting-drop vapour-diffusion crystallization experiments with hRab27a^Mut^(GppNHp) were set up using a Mosquito robot (TTP Labtech) at 20°C. A search for crystallization conditions was performed using ∼1000 commercial conditions. Drops consisting of 400 nl were formed by mixing equal volumes of protein solution and precipitant solution. The best crystals were obtained in 20%(*v*/*v*) ethylene glycol, 10%(*w*/*v*) PEG 8000, 30 m*M* MgCl_2_, 30 m*M* CaCl_2_, 100 m*M* HEPES pH 7.5 after 3–4 days at 20°C. Crystals were cryoprotected in the crystallization-condition solution supplemented with 30%(*v*/*v*) ethylene glycol and were flash-cooled in a nylon loop in liquid nitrogen. A complete X-ray data set to 2.82 Å resolution was collected at 100 K on beamline I02 at Diamond Light Source (DLS), Oxford, England. The data were processed and scaled with *DIALS* (Waterman *et al.*, 2016 ▸; Winter *et al.*, 2018 ▸), *POINTLESS* (Evans, 2011 ▸) and *AIMLESS* (Evans & Murshudov, 2013 ▸) using the *xia*2 pipeline (Winter, 2010 ▸). Statistics for the data collection are presented in Table 1 ▸. An initial model generated by molecular replacement with *Phaser* was refined through an iterative cycle using *Coot* (Emsley *et al.*, 2010 ▸) and *REFMAC*5 (Winn *et al.*, 2003 ▸). The final model structures were validated using the *MolProbity* server (Chen *et al.*, 2010 ▸) at http://molprobity.biochem.duke.edu. All structure images were prepared using *PyMOL* (Schrödinger).

A self-rotation function was calculated with *MOLREP* (Vagin & Teplyakov, 2010 ▸). Native Patterson maps were calculated with the *FFT* program (Ten Eyck, 1973 ▸) from the *CCP*4 package (Winn *et al.*, 2011 ▸). Anisotropic atomic displacement parameters, including the anisotropic delta-*B*, were calculated using the ANO (anisotropy) mode and tNCS expected intensity factors using the TNCS mode in *Phaser*. *SFTOOLS* from the *CCP*4 package (B. Hazes, unpublished results) was used to combine the anisotropy and tNCS factors, to select a subset of data for the initial structure solution and to compute the equivalent resolution corresponding to a full data set with a specified number of reflections. The Matthews coefficient (Matthews, 1968 ▸) and solvent-content calculations for different possible compositions of the asymmetric unit were carried out with *MATTHEWS_COEF* from the *CCP*4 package (Winn *et al.*, 2011 ▸).

## 3. Results and discussion   

### 3.1. Asymmetric unit composition and translational noncrystallographic symmetry   

The asymmetric unit of the hRab27a^Mut^(GppNHp) crystal was estimated to contain a large number of GTPase molecules (between 16 and 24; see Table 2 ▸; Kantardjieff & Rupp, 2003 ▸; Matthews, 1968 ▸; McCoy, 2007 ▸). With high NCS, the contribution of each component is small, making structure solution by MR much more challenging.

**Figure 1:**
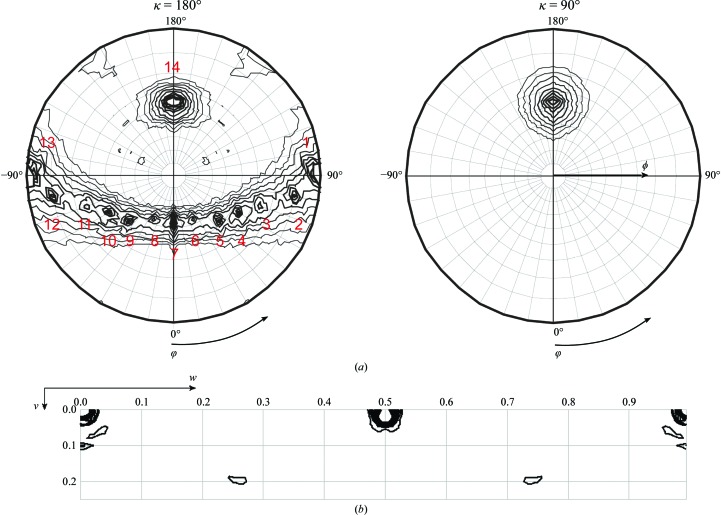
(*a*) Stereographic projection of the self-rotation function calculated for hRab27a^Mut^(GppNHp) crystals. The projections at κ = 180° and κ = 90° predict the presence of fourfold and twofold NCS axes (13 peaks on a slightly imperfect curved line in the plot, suggesting that the two pairs of tetramers are not exactly parallel) in the asymmetric unit. A full description of the labelled peaks is given in Table 3 ▸. (*b*) A slice of the Patterson map at *u* = 0 showing a strong off-origin peak at *v* = 0.022 and *w* = 0.500 with 45% of the height of the origin peak. This is a strong indicator of the presence of tNCS in the hRab27a^Mut^(GppNHp) crystals.

The self-rotation function reveals the angular relationship between two or more identical molecules in the asymmetric unit. This function measures the correlation of the native Patterson function with a rotated copy, often calculated using ω, φ and κ spherical polar angles. Self-rotation function peaks often correspond to rotational NCS in the crystal (Drenth, 2007 ▸). There is a κ = 90° (ω = [90°], φ = [54°]) peak in the self-rotation function (Fig. 1 ▸ *a*), corresponding to a fourfold rotation axis. There are also 13 κ = 180° peaks corresponding to twofold rotation axes. One interpretation of this is that there are two assemblies with dihedral *D*_4_ point-group symmetry in the crystal, with the two fourfold axes parallel.

Translational noncrystallographic symmetry (tNCS) occurs when two or more independent copies of a molecule have similar orientations in the unit cell. tNCS-related molecules would contribute with the same or similar amplitudes to a structure factor. However, their relative phases are determined by the projection of the translation vector on the diffraction vector, resulting in systematic interference that generates stronger and weaker reflections (Read *et al.*, 2013 ▸). This changes the usual Wilson distribution of structure-factor intensities (Read *et al.*, 2013 ▸; Wilson, 1949 ▸). The calculation of a native Patterson map for the hRab27a^Mut^(GppNHp) data reveals a peak at fractional coordinates (0.000, 0.022, 0.500) of 45% of the height of the origin peak (Fig. 1 ▸ *b*), showing strong tNCS that broadens the intensity distribution; because of the half-unit-cell component of the translation along the *c* axis, reflections with *l* odd will tend to be very weak, although this will be modulated by the size of the *k* index (because of the small but not insignificant translation along the *b* axis).

### 3.2. Extreme diffraction anisotropy   

**Figure 2:**
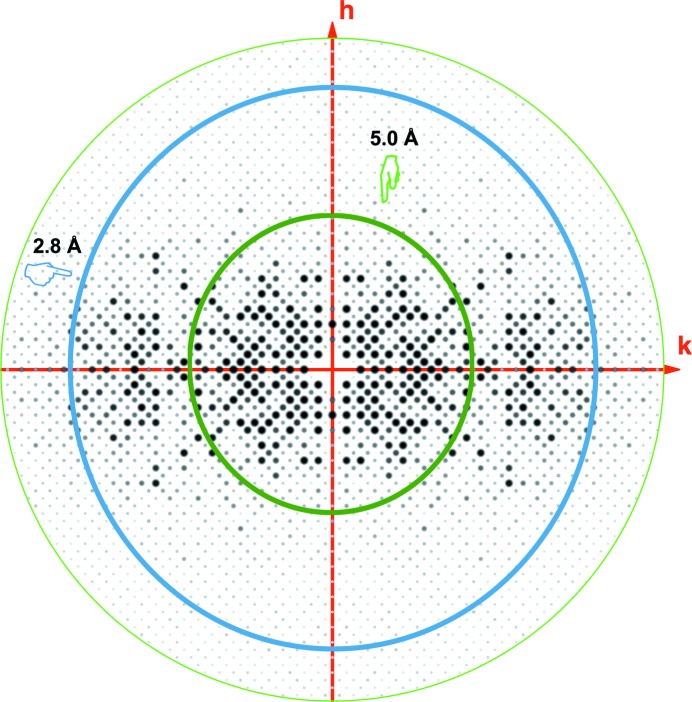
Pseudo-precession image of *hk*0 and image showing severe anisotropy in the data set, with the crystal diffracting to about 5.0 Å resolution in one dimension and 2.8 Å resolution in the other direction.

The hRab27a^Mut^ diffraction pattern was extremely anisotropic (Fig. 2 ▸), with the diffraction intensity falling off at different rates in different reciprocal-lattice directions. This is potentially owing to the pattern of lattice contacts in the crystal, which can give variations in the relative ordering of molecules along different directions. If not accounted for, the presence of significant anisotropy in the data will affect the likelihood functions used by *Phaser*, so it is important to refine and apply anisotropic correction factors. The degree of anisotropy of an X-ray data set can be described using the anisotropic delta-*B*, which is the difference between the two most extreme principal components of the anisotropic atomic displacement parameter along different directions in reciprocal space. Delta-*B* values of above 50 Å^2^ are considered to indicate extreme anisotropy. The diffraction anisotropy of the hRab27a^Mut^ crystals was estimated with the ANO mode of *Phaser* to be 207.3 Å^2^. This indicates a case of severe diffraction anisotropy (Fig. 2 ▸ *a*), with an effective resolution of 2.82 Å in the strongest direction and 5.0 Å in the weakest direction (Fig. 2 ▸ *b*).

### 3.3. Solving the molecular-replacement problem   

**Figure 3:**
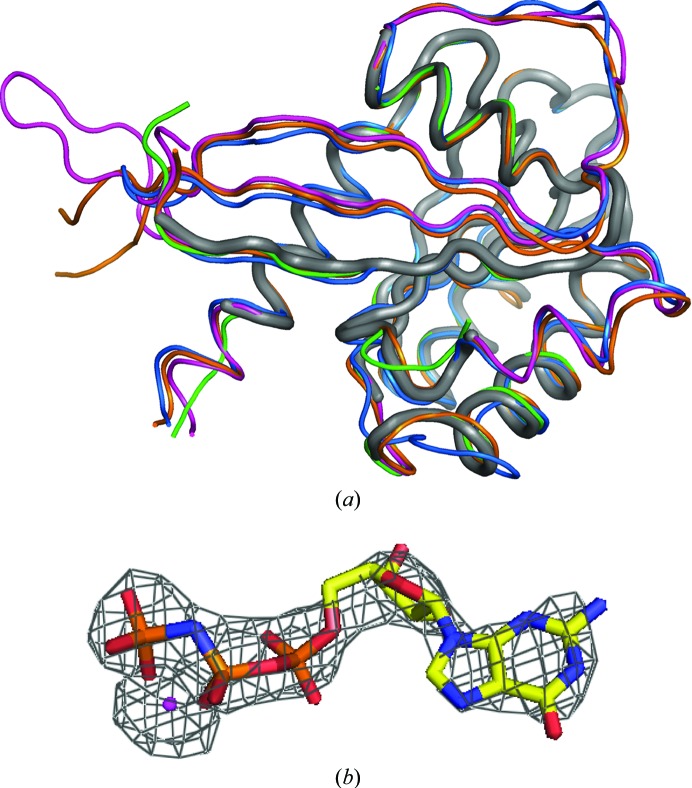
Solution of the hRab27a^Mut^(GppNHp) structure by MR. (*a*) Superposition of the trimmed ensemble used as the MR model (thick grey tube) with the untrimmed models used to generate it: mRab27a(GppNHp) (PDB entry 3bc1; magenta), mRab27b(GDP) (PDB entry 2iey; green), mRab27b(GppNHp) (PDB entry 2zet; orange) and human Rab8a(GppNHp) (PDB entry 4lhw; blue). (*b*) Detail of the *F*_o_ − *F*_c_ electron-density map (σ = 2.5) corresponding to the GppNHp molecule (in sticks) and the magnesium cation (magenta sphere).

After failed attempts to solve the structure with *Phaser* using the structure of mRab27a as a model, we used *Sculptor* (Bunkóczi & Read, 2011*a* ▸) and *Ensembler* (Bunkóczi & Read, 2011*b* ▸) to generate an optimized ensemble model. This ensemble was generated on the basis of the closest homologue structures reported for hRab27a: mRab27a(GppNHp) (PDB entry 3bc1; 87% identical in amino-acid sequence; Chavas *et al.*, 2008 ▸), mRab27b(GDP) (PDB entry 2iey; 68% identical; Chavas *et al.*, 2007 ▸), mRab27b(GppNHp) (PDB entry 2zet; Kukimoto-Niino *et al.*, 2008 ▸) and human Rab8a(GppNHp) (PDB entry 4lhw; 49% identical; Guo *et al.*, 2013 ▸). Regions with different conformations among the input models were removed using the ‘trim’ option of *Ensembler* (Fig. 3 ▸ *a*). A MR calculation with *Phaser* using this ensemble failed in the first attempt, where a solution was found for only one pair of tNCS-related copies.

**Figure 4:**
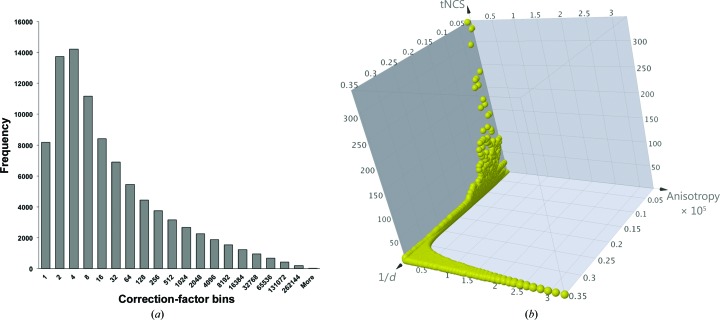
Frequency distribution of correction factors. (*a*) The frequency distribution of combined correction factors, binned on a logarithmic scale. (*b*) Anisotropy correction factors reach values about two orders of magnitude higher than those corresponding to tNCS. Moreover, while tNCS effects are limited to low resolution, anisotropy corrections predominantly affect high-resolution data. As a result, the two effects are uncorrelated (correlation coefficient of −0.02).

It appears that the combination of strong tNCS and extremely high anisotropy led to a very wide distribution of expected intensities, with many reflections expected to have extremely weak intensities based on these systematic effects. In addition, the high number of molecules in the asymmetric unit is likely to complicate the rotation and translation search functions. In principle, the new intensity-based likelihood target in *Phaser* (Read & McCoy, 2016 ▸) should compensate for the effects of anisotropy and tNCS by downweighting the systematically weak reflections with standard deviations that are large compared with their expected intensities. However, there could potentially be significant errors in the estimates of the standard deviations, as well as in the anisotropy and/or tNCS correction factors applied to the expected intensities. In addition, the presence of reflections with standard deviations much larger than their expected intensities could lead to numerical instabilities in the evaluation of the intensity-based likelihood target. To avoid these potential problems, the systematically weakest reflections with the largest relative errors were omitted from the molecular-replacement calculations. The anisotropic scale factors and tNCS scale factors were calculated using the ANO (anisotropy) and TNCS modes, respectively, in *Phaser*. Using *SFTOOLS*, these correction factors were multiplied together and then used to discard the systematically weakest intensities. In the initial calculation with the pruned data, any reflection for which the combined correction factor was greater than 10 was discarded; as a result, around 40% of the data were discarded (Fig. 4 ▸). Although both tNCS and anisotropy are present, for this data set by far the largest corrections arise primarily from the effects of anisotropy. The correction factors for anisotropy vary by a factor of nearly 330 000, while those for tNCS vary by a factor of less than 700, combining to give an overall variation by a factor of about 900 000 (Fig. 4 ▸). Note that the largest effects of tNCS are seen at low resolution, where small rotations and conformational differences have less effect on the correlations between the structure-factor contributions of tNCS-related molecules, while the largest effects of anisotropy are seen at high resolution; as a result, the range of the combined effects of tNCS and anisotropy is smaller than one would expect if the two effects varied independently.

Using the trimmed data, a clear and correct molecular-replacement solution could be found with a TFZ score of 12.8 for the final copy, placing 16 copies of the trimmed ensemble model in a physically plausible crystal-packing arrangement (Fig. 3 ▸ *b*); solutions with a TFZ of greater than 8 are almost always correct (Oeffner *et al.*, 2013 ▸). Testing different thresholds for the scaling-factor cutoff suggested that a 50× scaling-factor cutoff still gave an equivalent MR solution, enabling us to cut only 20% of the reflections. Density for the nucleotide, which was not included in the model, was observed in the NCS-averaged 2*F*_o_ − *F*_c_ and *F*_o_ − *F*_c_ electron-density maps (Fig. 3 ▸ *c*), strongly suggesting that the molecular-replacement solution was correct.

**Figure 5:**
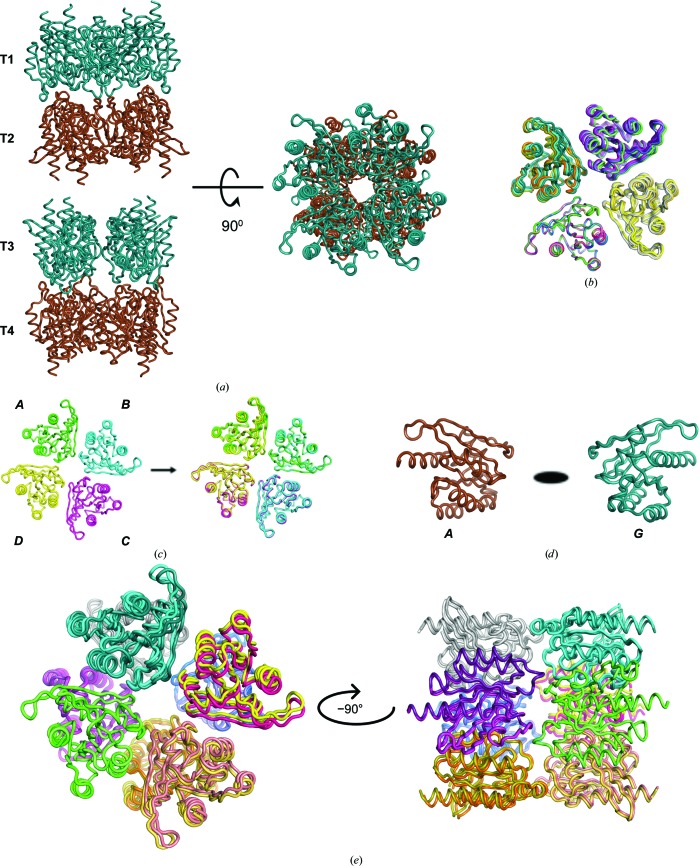
tNCS in the asymmetric unit found in the hRab27a^Mut^(GppNHp) crystals. (*a*) Asymmetric unit composition, consisting of four tetramers (T1–T4) represented as blue and brown ribbons. (*b*) Superposition of the four tetramers, showing that they share the same structure. (*c*) Each tetramer has a noncrystallographic fourfold axis, illustrated by superimposing molecule 1 (chain *A*) on molecule 2 (chain *B*) within a tetramer. (*d*) Pairs of molecules between pairs of tetramers are related by twofold symmetry axes: for example chain *A* (from T1) and chain *G* (from T2). (*e*) The superposition of T1 and T2 tetramers related by tNCS with T3 and T4 is shown.

The solution is also consistent with the self-rotation function. The asymmetric unit consists of two octamers, giving two *D*_4_ assemblies that superpose with very low r.m.s.d. values (<0.1 Å) using molecules *A* and *I* of T1 and T3, indicating that they have the same conformation/structure (Figs. 5 ▸ *a* and 5*b*). The fourfold axis of the octamer correlates with the peak in the self-rotation function at κ = 90° (κ = 90°, φ = ±180°, ϕ = 54°) and κ = 180° (κ = 180°, φ = ±180°, ϕ = 54° for the twofold axis within the same tetramer) (Fig. 1 ▸). The twofold axes relating molecules in one tetramer to molecules within other tetramers explain the peaks observed in the self-rotation function at κ = 180°. The peaks labelled 1–13 correlate to twofold axes between molecules in T1–T3, T1–T4, T2–T3 and T3–T4 (Fig. 1 ▸). A full description of the relationships is given in Table 3 ▸. In agreement with the prominent off-origin peak in the native Patterson map, translational symmetry between the two octamers is observed in the structure (Fig. 5 ▸ *e*).

The structure was completed and refined using *Coot* for manual rebuilding and *REFMAC*5 for refinement, during which noncrystallographic symmetry restraints were applied. Most residues in all 16 molecules were modelled, apart from flexible residues at the N-terminus of the construct. Residues with poor side-chain density (930 out of a total of 2736 in the model) were truncated at the C^β^ atom. The final refinement used a pruned data set from which reflections conveying less than 0.05 bits of information (24% of the data set) were removed, as discussed below. The agreement with the measured data (*R*_free_ = 0.342 and *R*_work_ = 0.312) is consistent with what one might expect from a data set containing 69 568 reflections; this corresponds to the number of reflections that would be contained in a complete isotropic data set at a resolution of 3.09 Å. The coordinates and structure factors have been deposited in the wwPDB (Berman *et al.*, 2007 ▸) as PDB entry 6huf.

**Figure 6:**
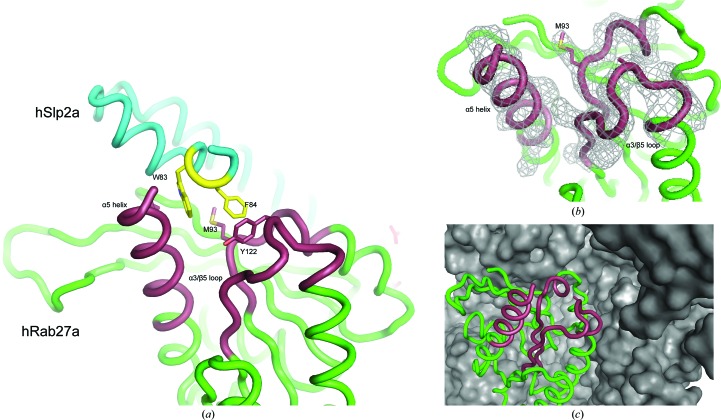
Accessibility of the SF4 pocket in the hRab27a^Mut^(GppNHp) crystals. (*a*) The interaction between hRab27a (green) and the hSlp2a Rab-binding domain (cyan). The SF4 pocket (red), comprised of the α3/β5 loop and the α5 helix, interacts with the WF motif (yellow) of hSlp2a. (*b*) The SF4 pocket is well defined in the structure of the hRab27a^Mut^(GppNHp) crystals. (*c*) The pocket is not occluded by neighbouring molecules (shown in surface representation) and all copies in the asymmetric unit are accessible for ligand-interaction studies.

In the Rab27a structure, the SF4 pocket, formed by the α3–β5 loop (a highly variable region among Ras superfamily members) and the C-terminal region of the α5 helix, is of particular interest, as it is fundamental to the interaction of Rab27a with the WF motif of Slp2a. A model was built for the SF4 pocket in all 16 molecules of the solution structure. Interestingly, the pocket is free from contacts with neighbouring symmetry-related molecules (Fig. 6 ▸), making it suitable for protein–ligand interaction studies if the problems with anisotropy in the data could be resolved.

### 3.4. Excluding systematically weak data based on information content   

Subsequent to, and inspired by, this structure solution, an automated method to exclude the systematically weakest reflections from the MR likelihood calculations has been implemented in *Phaser*. The method applied in the initial structure solution was chosen to eliminate the reflections that would suffer most from the combined effects of anisotropy and tNCS, but it did not account for the precision of the individual measurements.

The new method considers the precision of the measurement relative to the intensity expected for the particular reflection when the effects of anisotropy and tNCS are taken into account. One way to evaluate the precision of a measurement is to consider how much information that measurement conveys; in other words, how much more is known after making the measurement than before. This information gain can be evaluated by considering the loss of relative entropy in going from the prior probability distribution [the null hypothesis, in this case the Wilson (1949 ▸) distribution of true intensities] to the posterior probability distribution. In information theory, this quantity is known as the Kullback–Leibler divergence or KL-divergence (Kullback & Leibler, 1951 ▸), which is defined in (1) and is represented subsequently as simply *D*_KL_:
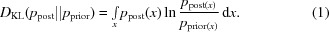
If the natural logarithm is used in this expression, the information content is expressed in units of nats, whereas the equivalent expression using the base 2 logarithm gives information in terms of bits, which can therefore be obtained from that in nats by dividing by ln(2). The KL-divergence is always non-negative, but because the integral is weighted by only one of the two probability distributions it is not symmetric and is therefore not strictly a distance metric.

This information-based measure is a natural choice in the context of likelihood-based optimization methods. If in the KL-divergence in (1) the prior probability is replaced by a prior probability conditional on a model, then it can be shown that maximizing a likelihood function (*i.e.* the probability of the data given the model) is equivalent to minimizing this KL-divergence (Bishop, 2006 ▸). In other words, maximizing the likelihood minimizes the divergence between the probability of the true value of the data given the model and the probability of the true value of the data given the measurements of the data.

For diffraction data measured in terms of intensities and their estimated standard deviations, the expressions are simpler if cast in terms of normalized intensity values, for which the expected true intensity is 1, *i.e.* *E* ^2^. For clarity, we will represent the normalized intensity as *Z* (= *E* ^2^). The prior probability is simply the Wilson (1949 ▸) distribution of normalized intensities, given in (2*a*) for the acentric case and (2*b*) for the centric case:
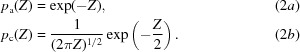



In computing the KL-divergence for diffraction intensities, the posterior probability of the true intensity given the measured intensity, which plays a key role in the procedures of French & Wilson (1978 ▸), can be defined in terms of other probabilities using Bayes’ theorem (3), yielding (4):
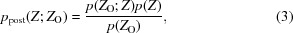


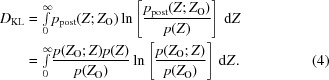
In this equation, the probability distribution for the observed intensity given the true intensity is taken as the Gaussian distribution in (5), 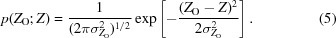
The probability distribution for the observed normalized intensity is given by (6*a*) for acentric reflections and by (6*b*) for centric reflections, which are reproduced from equations (9*a*) and (9*b*) of Read & McCoy (2016 ▸):
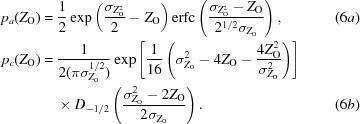



In (6*b*), erfc is the complement of the error function and *D* is a parabolic cylinder function (Whittaker & Watson, 1990 ▸).

The integral in (4) could be used to evaluate the information content of individual reflections, and a minimum information content could be defined for reflections that are accepted for further calculations. We chose instead to evaluate and use the *expected* value of the information content, based only on the estimated standard deviation and ignoring the particular value found for the measured intensity. The primary argument for this choice is that outlier observations are probably more likely to be encountered for the systematically weak intensities, partly because of inaccuracies in the determination of the correction factors; outliers that are substantially larger than expected will be evaluated, according to (4), as conveying more information and would thus be more likely to be kept in the data set. An additional advantage to using the expected information content is that this is a function of only the standard deviation of the normalized intensity, so a simple threshold can be set. In contrast, evaluating the integral in (4) is considerably more difficult, but in the future we will test whether there is a practical difference in outcome.

The expected information content is a probability-weighted average over all possible values of the measured intensity, given in (7):
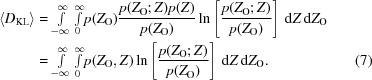



The derivation of (7) implicitly assumes that the standard deviation of the intensity is independent of the measured intensity, which would not be valid for well measured intensities. However, the information thresholds are only applied in practice to observations in which the uncertainty of the measurement is at least several times larger than the expected intensity itself (see below); in these circumstances the uncertainty comes primarily from the counting statistics of the background rather than the peak.

To construct lookup tables for normalized intensity standard deviation thresholds, (7) was evaluated by numerical integration in *Mathematica* v.10 (Wolfram Research, Champaign, Illinois, USA) for a variety of expected information-content thresholds. Information-content filtering based on these thresholds was implemented in *Phaser* (McCoy *et al.*, 2007 ▸), with the feature being available in v.2.7.17 (November, 2016) or newer. Note that the systematically weak reflections contribute to the refinement of parameters describing the anisotropy and tNCS, and are only excluded for subsequent MR likelihood calculations; for this reason, it is better to provide the full, unpruned set of data to *Phaser*.

An examination of (7) gives further insight into the connection between the KL-divergence and likelihood. The form of this equation is highly reminiscent of the expected log-likelihood gain (eLLG) used to predict the outcome of molecular-replacement calculations, as defined in equation (3) of McCoy *et al.* (2017 ▸). This equation can be recast in terms of observed intensities rather than effective amplitudes, yielding (8),
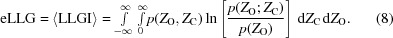
For the case of a perfect model, where the calculated structure factor is identical to the true structure factor, this equation for the eLLG is equivalent to the expected KL-divergence. In other words, the expected KL-divergence corresponds to the estimated maximum contribution of an observation to the total likelihood that could be achieved with a perfect model.

### 3.5. Accounting for measurement error in the likelihood-based fast rotation function   

Inspection of the log files obtained in the initial structure solution before and after pruning the data with the largest anisotropy and tNCS correction factors suggested that the greatest improvements from omitting systematically weak data were in the results of the fast rotation function. This revealed an oversight in the implementation of the intensity-based LLGI target function in *Phaser* (Read & McCoy, 2016 ▸). In almost all cases, implementing this target simply involves replacing the structure-factor amplitude with an effective amplitude, *F*_eff_, and applying an additional factor *D*_obs_ to any σ_A_ values in the likelihood targets; both *F*_eff_ and *D*_obs_ are derived from the intensity and its standard deviation (Read & McCoy, 2016 ▸).

Applying this to the likelihood-based fast rotation function, LERF1 (Storoni *et al.*, 2004 ▸), requires a slightly different approach. LERF1 is based on a first-order series expansion of the log of the rotation likelihood function, given in (9) (adapted from equation 17 of Storoni *et al.*, 2004 ▸),
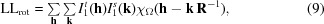
where χ_Ω_ is the Fourier transform of the sphere inside of which Patterson-like functions of the observed intensities and contributions of the fixed and rotating components of the model are compared as a function of rotation. (Note that the post-multiplication of **k** by **R** ^−1^ corresponds in reciprocal space to rotating the calculated Patterson in direct space by pre-multiplying the coordinates by **R**.) The Patterson-like functions *I*_1_^*t*^ and *I*_1_^*s*^ are defined in (10*a*)–(10*c*), which are adapted from equations (18) and (19) of Storoni *et al.* (2004 ▸):
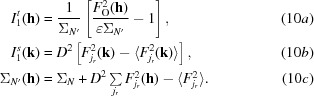



In (10*b*) and (10*c*) *D* is the Luzzati factor (Luzzati, 1952 ▸), which is proportional to σ_A_. In the initial adaptation of LERF1 to the LLGI intensity-based likelihood target, any instances of *D* in the variance term Σ_*N*′_ in (10*a*) were multiplied by *D*_obs_. However, the Luzzati factor *D* in (10*b*) was not modified, because rotation of the model associates different indices **k** with the observed reflections indexed by **h**. To compensate in (9) for this omission, the expression for *I*_1_^*t*^ has to be multiplied by *D* ^2^_obs_. This correction was introduced into *Phaser* at the same time as the filtering on information content.

### 3.6. Tests of modified *Phaser*   

As described above, eliminating the systematically weakest reflections from the data set was sufficient to give a clear solution to the hRab27a structure, even before the fast rotation function was modified to properly account for intensity-measurement errors.

With the new algorithms, the hRab27a structure and others suffering from severe anisotropy and/or tNCS can now be solved more easily and without manual intervention. Table 4 ▸ illustrates the effect of applying different information-content thresholds on the course of the molecular-replacement calculation. With the corrected fast rotation function, it is no longer necessary to prune the systematically weak reflections in order to obtain a solution. Pruning up to about 19% of the weakest reflections in this data set (those conveying less than 0.01 bits of information each) has very little effect on the signal; if anything, the final LLG value increases very slightly. For this case at least, there is very little disadvantage to including even exceptionally weak data as long as the effects of measurement errors are accounted for properly. The main effect is a tendency for the total computing time to increase with the number of reflections included. (Note that there is a stochastic element to the total computing time, which is influenced by the number of potential partial solutions identified at any point in the calculation.) For other cases, where the estimates of measurement errors might be poorer or where the effects of anisotropy and/or tNCS might be modelled less accurately, omitting the weakest reflections might still improve the outcome of the calculation.

However, our experience with the oversight in the implementation of the fast rotation function shows that when an algorithm fails to account properly for the effects of measurement error, there is a real advantage to pruning the weakest data. In the uncorrected fast rotation function, terms corresponding to weak observations with little information content were being included at a higher weight than they should have been given. The same general effect will apply in any other calculation in which weak data are not appropriately downweighted. For instance, the use of amplitudes and their standard deviations obtained through the French & Wilson (1978 ▸) algorithm in amplitude-based refinement likelihood targets will overweight extremely weak data because the French and Wilson amplitude standard deviation has a finite value even in the limit of intensities with infinite measurement error (Read & McCoy, 2016 ▸).

The relationship between the expected LLG and the expected KL-divergence (equations 7 and 8) shows that even for a model approaching perfection, the omission of data with low information content will have very little effect on a properly calculated likelihood function, indicating that such observations should have very little leverage. For instance, measurements contributing 0.01 bits of information will contribute at most 0.01ln(2) to the likelihood score, so it would take over 140 such observations to change the likelihood score by a single unit. If such observations are omitted from algorithms in which the effects of errors are not properly accounted for, this will remove a potential source of systematic bias or noise.

The expected information content could therefore potentially be used as an alternative to ellipsoidal truncation to prune weak data (Strong *et al.*, 2006 ▸). The initial approach, that of pruning the reflections with the highest combined anisotropy and tNCS correction factors, led to a successful structure solution but does not work nearly as well. For instance, if the 23 629 reflections with a combined intensity-correction factor of greater than 60 are omitted, the final LLG decreases from 3667.3 to 3560.2, whereas if the 23 868 reflections conveying less than 0.1 bits of information are omitted the final LLG only decreases to 3646.8. As a less extreme example, 17 457 reflections have a combined correction factor of greater than 160; if these are omitted the final LLG decreases to 3659.3, whereas setting the information-content threshold to 0.01 bits actually gives a slight increase in LLG while omitting a very similar number of reflections (Table 4 ▸).

Based on these data and similar tests on other systems (results not shown), the default threshold chosen for likelihood calculations in *Phaser* is 0.01 bits of information per reflection; note that all data should be used in the data-preparation calculations in *Phaser* that characterize anisotropy and tNCS effects. Optimal thresholds for computations in other software are likely to differ from this. In addition, the information calculations depend on the accuracy of the parameters describing anisotropy and tNCS, and do not yet account for other effects on intensities such as those from twinning or order–disorder structures. The full data set should therefore always be maintained without permanently excluding data at any information threshold.

## 4. Conclusions   

The hRab27a^Mut^(GppNHp) data show how difficult cases of molecular replacement can be solved using *Phaser* if anisotropy and tNCS are properly accounted for using strategies that are applied automatically in *Phaser* v.2.7.17 or newer. Moreover, the structure of the hRab27a^Mut^(GppNHp) crystals shows that the SF4 pocket, which is the primary target for ligand-binding studies, is unoccupied and could be used to study the structure of ligands binding to Rab27a. The only major drawback is the data quality, specifically the overall resolution and severe anisotropy, which would be problematic for weak binding ligands with low occupancy. Optimization of crystallization conditions, additive screens and the structure of hRab27a^Mut^(GppNHp) reported here will guide further construct design to obtain a more tractable crystal form for ligand-binding studies.

## Supplementary Material

PDB reference: human Rab27a, 6huf

## Figures and Tables

**Table 1:** Data-collection statistics for hRab27a^Mut^(GppNHp) crystals Values in parentheses are for the highest resolution shell.

Crystal data	
Space group	*C*2
Unit-cell parameters	
*a* (Å)	130.35
*b* (Å)	132.42
*c* (Å)	230.42
β (°)	103.52
Data collection	
Beamline	I02, DLS
Detector	PILATUS 6M-F
Total oscillation (°)	200
Oscillation per image (°)	0.2
Wavelength (Å)	0.97949
Temperature (K)	100
Resolution (Å)	57.00–2.82 (2.88–2.82)
Total No. of reflections	338434 (25355)
No. of unique reflections	91204 (4512)
Multiplicity	3.7 (5.6)
Half-data-set correlation coefficient CC_1/2_	0.995 (0.688)
Completeness (%)	99.9 (100.0)
〈*I*/σ(*I*)〉	7.9 (1.0)
*R*_merge_ †	0.081 (0.991)
*R*_meas_ ‡	0.109 (1.038)
*R*_p.i.m._ §	0.073 (0.691)
Data statistics for truncated data	
Resolution (Å)	57.00–2.82 (2.88–2.82)
Completeness (%)	75.8
〈*I*/σ(*I*)〉	10.3 (2.1)
Refinement statistics for truncated data	
Reflections used in refinement	66055 (2950)
Reflections used for *R*_free_	3513 (170)
*R*_work_	0.312 (0.465)
*R*_free_	0.342 (0.490)
No. of non-H atoms	
Total	18977
Macromolecules	18443
Ligands and waters	534
Protein residues	2736
R.m.s.d., bonds (Å)	0.005
R.m.s.d., angles (°)	1.48
Ramachandran favoured (%)	91.1
Ramachandran allowed (%)	7.8
Ramachandran outliers (%)	1.1
Rotamer outliers (%)	0.1
Clashscore	10.7
*B* factors (Å^2^)	
Average	75.0
Macromolecules	84.7
Ligands	56.9
Waters	37.8

†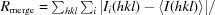 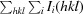.

‡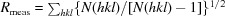 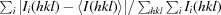.

§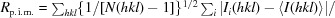 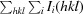.

**Table 2:** Estimation of the number of molecules in the asymmetric unit for the hRab27a^Mut^(GppNHp) crystals *N*_mol_, number of molecules; *P*, probability. The correct composition is highlighted in bold.

*N*_mol_ in asymmetric unit	Matthews coefficient (Å^3^ Da^−1^)	Solvent content (%)	*P* (2.82 Å)	*P*(tot)
11	4.34	71.7	0.00	0.00
12	3.98	69.1	0.01	0.01
13	3.68	66.6	0.02	0.01
14	3.41	64.0	0.03	0.02
15	3.19	61.4	0.04	0.03
**16**	**2.99**	**58.8**	**0.06**	**0.05**
17	2.81	56.3	0.08	0.07
18	2.65	53.7	0.11	0.10
19	2.52	51.1	0.12	0.12
20	2.39	48.6	0.13	0.13
21	2.28	46.0	0.13	0.13
22	2.17	43.4	0.11	0.12
23	2.08	40.8	0.08	0.09
24	1.99	38.3	0.04	0.06
25	1.91	35.7	0.02	0.03
26	1.84	33.1	0.01	0.01

**Table 3:** Assignment of peaks corresponding to a twofold axis between molecules on the κ = 180° self-rotation function map

Peak No. at κ = 180°	Polar angles	Related molecules
1	κ = 180°, φ = 90°, ϕ = 88°	*D*–*P*, *C*–*M*, *H*–*L*, *F*–*J*
2	κ = 180°, φ = 80°, ϕ = 80°	*I*–*O*, *L*–*P*, *K*–*M*, *J*–*N*
3	κ = 180°, φ = 73°, ϕ = 63°	*A*–*H*, *B*–*G*, *D*–*E*, *C*–*G*
4	κ = 180°, φ = 60°, ϕ = 54°	*A*–*P*, *B*–*O*, *D*–*M*, *C*–*N*, *E*–*L*, *H*–*I*, *F*–*K*, *G*–*J*
5	κ = 180°, φ = 46°, ϕ = 45°	*I*–*P*, *L*–*M*, *K*–*N*, *J*–*O*
6	κ = 180°, φ = 25°, ϕ = 37°	*A*–*E*, *B*–*H*, *D*–*F*, *C*–*F*
7	κ = 180°, φ = 0°, ϕ = ±35°	*A*–*M*, *B*–*P*, *D*–*N*, *C*–*O*, *E*–*I*, *H*–*J*, *F*–*L*, *G*–*K*
8	κ = 180°, φ = −25°, ϕ = 37°	*I*–*M*, *L*–*N*, *K*–*O*, *J*–*P*
9	κ = 180°, φ = −45°, ϕ = 46°	*A*–*F*, *B*–*E*, *D*–*G*, *C*–*H*
10	κ = 180°, φ = −60°, ϕ = 55°	*A*–*N*, *B*–*M*, *D*–*O*, *C*–*P*, *E*–*J*, *H*–*K*, *F*–*I*, *G*–*L*
11	κ = 180°, φ = −73°, ϕ = 64°	*I*–*N*, *L*–*O*, *K*–*P*, *J*–*M*
12	κ = 180°, φ = −80°, ϕ = 80°	*A*–*G*, *B*–*F*, *D*–*H*, *C*–*E*
13	κ = 180°, φ = −90°, ϕ = 88°	*A*–*O*, *B*–*N*, *E*–*K*, *G*–*I*
14	κ = 180°, φ = ±180°, ϕ = 54°	*A*–*C*, *B*–*D*, *E*–*G*, *F*–*H*, *I*–*K*, *J*–*L*, *M*–*O*, *N*–*P*

**Table 4:** Effect of expected information-content thresholds on molecular replacement

	σ(*Z*) threshold				
Threshold (bits)	Centric	Acentric	Final LLG	CPU (s)	No. of reflections omitted† (%)
None	—	—	3667.3	5627	8‡ (0.0%)
0.001	37.96	26.84	3666.8	5240	12857 (14.1%)
0.005	16.95	11.99	3668.7	4574	15952 (17.5%)
0.01	11.97	8.46	3669.1	5016	17477 (19.2%)
0.05	5.26	3.73	3661.5	4789	21622 (23.7%)
0.1	3.61	2.58	3646.8	4970	23868 (26.2%)

† From a total of 91 204 reflections.

‡ Reflections rejected as Wilson distribution outliers.
